# Neuromuscular and balance adaptations following acute stretching exercise: a randomized control trial

**DOI:** 10.3389/fphys.2024.1486901

**Published:** 2024-12-03

**Authors:** Bin Wang, Bin Wu, Yi Yang, Mengbiao Cai, Shewang Li, Hui Peng

**Affiliations:** ^1^ Department of Physical Education, Nanjing Vocational College of Information Technology, Nanjing, China; ^2^ Department of Physical Education, Nanjing City Vocational College, Nanjing, China; ^3^ College of Physical Education, Hengxing University, Qingdao, China; ^4^ Department of Physical Education, Nanjing University of Aeronautics and Astronautics, Nanjing, China; ^5^ Department of Basic Courses, Suzhou City University, Suzhou, China; ^6^ School of Wushu and Chinese Traditional Sports, Tianjin University of Sport, Tianjin, China

**Keywords:** neuromuscular, balance, stretching, exercise, adaptation

## Abstract

**Purpose:**

This study aimed to examine the acute effects of static stretching (SS) and dynamic stretching (DS) on neuromuscular function and balance in recreationally active men.

**Method:**

Sixty participants were randomly assigned to SS, DS, or no stretching (NS) groups. Before and after their respective stretching protocols, participants were assessed using the stork balance test (SBT), Y-balance test (YBT), T-change of direction test (CoD T-test), countermovement jump test (CMJT), squat jump test (SJT), and five-time jump test (FJT).

**Results:**

Significant main effects of time were observed for the SBT, YBT, and CoD T-test. Significant interactions between time and group were found for the SBT, YBT, CoD T-test, and CMJT (P < 0.05). Compared to the NS group, the SS group showed significant improvement in the SBT (P < 0.05), while the DS group demonstrated significant improvements in the SBT, YBT (all directions), CoD T-test, CMJT, and SJT (P < 0.05). Post-training, the DS group showed greater improvements than the SS group in the YBT, CoD T-test, CMJT, and SJT (P < 0.05), with no significant differences in the SBT.

**Conclusion:**

SS acutely improves static balance, while DS has a broader impact, enhancing both neuromuscular function and balance.

## 1 Introduction

Stretching exercises are usually performed as part of a warm-up routine to prepare the body for physical activity and enhance exercise performance. These exercises encompass various types including static and dynamic stretching, each offering distinct benefits and potential drawbacks (Behm and Chaouachi, 2011). Static stretching involves holding a muscle at its maximal length for a prolonged period and repeating this process, enhancing muscle flexibility, stretchability, and range of motion (Curry et al., 2009; McHugh and Cosgrave, 2010; Behm and Chaouachi, 2011). In contrast, dynamic stretching involves active movements that mimic the motions of the upcoming activity, promoting muscle activation and functional flexibility (Little and Williams, 2006; Curry et al., 2009).

The benefits of both static and dynamic stretching have been well-documented, including increased muscle compliance, reduced resistance to stretch, improved performance outcomes such as sprinting speed, and decreased injury risk during physical activities (Herbert and Gabriel, 2002; McHugh and Cosgrave, 2010; Franco et al., 2012). Despite these advantages, the acute effects of stretching on neuromuscular adaptations and balance control remain areas of ongoing investigation.

From the perspective of neuromuscular adaptation, different types of stretching elicit distinct effects. Research indicates that static stretching may lead to a temporary reduction in muscle power and performance, particularly in activities that require explosive movements (Kistler et al., 2010; McHugh and Cosgrave, 2010; Behm and Chaouachi, 2011). In contrast, dynamic stretching generally results in more positive effects, enhancing maximal strength and sprint performance (Yamaguchi and Ishii, 2005; Curry et al., 2009). The mechanisms by which static and dynamic stretching influence neuromuscular adaptation differ fundamentally. Static stretching affects neuromuscular function by altering muscle tension and nerve conduction velocity (McHugh and Cosgrave, 2010), whereas dynamic stretching engages both the muscles and the nervous system, enhancing motor reflexes and neuromuscular coordination (Behm and Chaouachi, 2011; Coratella et al., 2021). Despite this, existing studies mostly focus on the isolated effects of static or dynamic stretching, with relatively few comparing the two directly in terms of neuromuscular adaptation. A direct comparison of the neuromuscular effects of static versus dynamic stretching across individuals with varying levels of athletic ability could provide valuable insights into the strengths and limitations of each approach.

From the perspective of balance adaptation, the effects of static stretching are more complex (Coratella et al., 2021a)). Some studies suggest that static stretching can improve flexibility and joint range of motion, potentially enhancing balance (Behm and Chaouachi, 2011; Fowles et al., 2000). However, other research indicates that static stretching may negatively affect balance, particularly in tasks requiring dynamic control and quick responses (Simic et al., 2013; Kay and Blazevich, 2012). This adverse effect may be due to static stretching’s influence on muscle tension and nervous system response, which can reduce muscle explosiveness and reaction time, thus impairing the ability to rapidly adjust balance (Herbert and de Noronha, 2007; He and Fekete, 2021). In contrast, dynamic stretching, with its varied range of motion and speed, activates a broader set of muscle groups and neural pathways, potentially improving the body’s adaptability in complex balance tasks (Behm and Chaouachi, 2011). It is important to note that both static and dynamic stretching’s effects on balance are influenced by the timing, intensity, and duration of the stretch (Kay and Blazevich, 2012). Consequently, exploring the specific impact of different static or dynamic stretching protocols on balance adaptation holds significant practical relevance for optimizing training and performance.

In summary, this study aims to assess and compare the acute effects of a carefully designed static stretching protocol and a dynamic stretching protocol on neuromuscular and balance adaptability in recreationally active men. The static stretching protocol consists of 10 stretches targeting specific muscle groups, with each stretch performed for 1 to 2 sets, each lasting 30 s. The dynamic stretching protocol incorporates dynamic movements, controlled motions within the full range of motion, general exercise routines, and light bouncing exercises, and includes 13 stretches targeting the same muscle groups as the static protocol. The hypothesis of this study is as follows: static stretching improves neuromuscular adaptability but may negatively affect balance adaptability, while dynamic stretching positively influences both neuromuscular and balance adaptability.

## 2 Methods

### 2.1 Participants

Sixty male college students (Mean ± SD, age: 21.60 ± 1.76 years) were recruited for this study. Subjects were required to be recreationally active adults (Adults aged 18–64 years old completing at least 150–300 min moderate-intensity activity or 75–150 min of vigorous-intensity activity a week, plus muscle-strengthening activities 2 or more days a week) (Ayala et al., 2014) and have no cardiovascular disease, or any orthopedic injuries within the 6 months prior to testing. All subjects were briefed regarding potential risks and provided written informed consent forms. The study was performed in accordance with the Declaration of Helsinki and was approved by the Ethics Committee of Sports Science Experiment (TJUS2024-033).

### 2.2 Experimental protocols

A between-group design was selected for this study. Subjects were randomly divided into three groups using computer randomization: the static stretching group (SS group), dynamic stretching group (DS group), and no stretching group (NS group), each consisting of 20 individuals. The randomization was performed using a computerized algorithm with a random number generator to ensure equal distribution of 20 participants per group. The NS group served as a control. All three groups began with a general 10-min warm-up session involving light jogging at rating of perceived exertion of 3–5. Following this warm-up, all subjects rested for 3 min and performed the stork balance test (SBT), Y-balance test (YBT), T-change of direction test (CoD T-test), countermovement jump test (CMJT), squat jump test (SJT), and five-time jump test (FJT). After the rest period and initial assessments, the SS group and DS group performed their respective stretching protocols, each lasting approximately 8.5 min, while the NS group rested for 8.5 min. Subsequently, all subjects underwent post-intervention assessments, including the SBT, YBT, CoD T-test, CMJT, SJT and FJT. Additionally, participants received standardized guidance on each stretching movement from trained staff before and during the intervention to ensure consistent execution of the stretches.

### 2.3 Stretching programs

#### 2.3.1 Static stretching

The SS protocol was designed to be as close as possible to currently used pre-activity stretching while maintain standardization for research purposes, consisting of 10 stretching movements targeting specific muscle groups: gluteals, adductors, spinal erectors, abdominals, obliques, pectorals, iliotibial band, quadriceps, hamstrings, gastrocnemius, and soleus muscles. Each movement involved 1 or 2 sets, with each set lasting 30 s. This protocol was selected because SS durations of 2 min or more are frequently utilized in similar studies. Moreover, it has been reported that times longer than a single 30-s set do not yield further improvements in certain sports performance indicators. Table 1 presents the names of the movements, the targeted muscles, and the sequence of stretches performed by the SS group.

**TABLE 1:** Static stretching protocol.

Movement name	Targeted muscle	Sets	Time (s)
cross-leg stretch	gluteals	2 (1 per leg)	30*2
butterfly stretch	adductors	1	30
sit and reach	spinal erectors and hamstrings	1	30
floor back extension	abdominals	1	30
lateral bend	obliques	2 (1 per side)	30*2
wall pec stretch	pectorals	2 (1 per side)	30*2
abductor stretch	iliotibial band	2 (1 per leg)	30*2
standing 1-leg quadriceps stretch	quadriceps	2 (1 per leg)	30*2
seated 1-leg hamstring stretch	hamstrings	2 (1 per leg)	30*2
calf stretch	gastrocnemius and soleus muscles	2 (1 per leg)	30*2

#### 2.3.2 Dynamic stretching

The DS protocol was designed to streamline commonly used stretching techniques before practices, games, or competitions. It comprised 13 stretching movements focusing on lower extremity muscle groups, supplemented with active upper-extremity movements targeting the same muscle groups as the SS routine. Participants in the DS group engaged in a combination of mobilization activities, controlled movements across an active range of motion, general movement drills, and light plyometric exercises. Similar protocols are currently employed globally, such as the Parisi Warm-up Method, which has benefitted over 250,000 athletes across all competitive levels (34). Table 2 presents the names of the movements, the targeted muscles, and the number of repetitions performed by the DS group.

**TABLE 2:** Dynamic stretching protocol.

Movement name	Targeted muscle	Repetitions
supine knee rocking	gluteals, spinal erectors	20
prone scorpion	quadriceps, gluteus maximus, obliques	10
hand walkout (inchworm)	spinal erectors, gastrocnemius, soleus	5
prisoner squats	quadriceps, hamstrings, rhomboids	10
side-step squats	gluteus medius, quadriceps, hamstrings	10 per side
lunge with twist	quadriceps, hamstrings, obliques	5 per side
45° T-lunges	gluteus medius, quadriceps, hamstrings, rhomboids	4 per side
high knees	quadriceps, gastrocnemius, soleus	20
heel kicks	hamstrings, gluteus maximus, gastrocnemius, soleus	20
leg swing	hamstrings, illiopsoas, gluteus maximus	10 per side
box drill hops (counter and clockwise)	gastrocnemius, soleus	10
single leg hops (back and forth)	gastrocnemius, soleus	10 per side

### 2.4 Testing procedure and measures

All tests were undertaken by the same investigator to ensure the quality of the measurement results. Testings were performed in the following order: (a) SBT; (b) YBT; (c) CoD T-test; (d) CMJT and SJT; (e) FJT. A 2-min recovery period was provided between each test.

#### 2.4.1 Stork balance test

The SBT (for both right and left legs) was used to assess the static balance ability of the participants. During the tests, participants were instructed to place their hands on their hips, with non-supporting foot positioned on the inner knee of the supporting leg. They were then required to lift the heel of the supporting foot to maintain balance. Timing commenced as soon as the supporting heel left the floor. The test concluded if the supporting heel made contact with the ground, if the supporting foot moved or rotated, or if the non-supporting foot lost contact with the knee. Each leg of the participant was tested twice, and the optimal value was taken for data analysis.

#### 2.4.2 Y-balance test

The YBT was used to assess the dynamic balance ability of the participants. A YBT kit was utilized, consisting of a stance platform with three attached pipes extending in the anterior (AT), posteromedial (PM), and posterolateral (PL) directions. The posterior (PM and PL) pipes were positioned at 135° angles from the AT pipe, with 45° spacing between them. During the tests, participants were instructed to maintain single-leg balance while reaching as far as possible with the other leg in each of the three directions, and the maximum distance that they can stretch and return were measured. The participants underwent twice tests with a 2-min recovery period, and the optimal values were taken.

#### 2.4.3 T-change of direction test

The CoD T-test assessed agility and neuromuscular function through short-distance forward, lateral, and backward running. The test setup included four cones arranged in a T formation, and times were recorded using an electronic timing system (Brower Timing Systems, Salt Lake City, UT, United States) according to the established protocol. Participants perform sprints, lateral shuffles, and backpedals involving four directional changes, which represent typical neuromuscular movements. Each participant completed two tests with a 2-minute recovery period, and the fastest time recorded was selected for further analysis.

#### 2.4.4 Countermovement jump and squat jump test

The CMJ evaluates reactive strength of the lower limbs through an initial countermovement before the toe-off phase, while the SJ assesses leg power performance from a stationary, semi-squatting position (Petrigna et al., 2019). During the CMJT, participants were instructed to keep their hands on their waist, start from a standing position, and perform a preparatory counter-movement. In the SJT, participants were instructed to keep their hands on their waist, lower themselves to a 90° knee flexion position where the upper thighs are parallel to the ground, and held this position for 3 s before executing a maximal vertical jump without any preceding countermovement. CMJT and SJT were eacn repeated three times on a platform equipped with an optoelectrical system (Opto-Jump Microgate, Italy). Flight times were recorded using a digital timer connected to the platform, and these times were utilized to calculate jump height with the formula 1/8×g×t2 (g = gravity, t = time). The best jumps out of the three test was selected respectively for subsequent analysis.

#### 2.4.5 Five-time jump test

The FJT is often used to assess lower limb muscle power, involving 5 consecutive strides with joined feet position at the start and end of the jumps. In the test, participants began with a parallel foot position, alternated jumping with their left and right feet twice, and concluded with a final jump where their feet returned together. Test performance was measured in meters, rounded to the nearest centimeter. Each participant underwent two test sessions separated by a 2-minute recovery period, and the best result was recorded for analysis.

### 2.5 Statistical analyses

The experimental data were analyzed using IBM SPSS statistical software package (version 26.0, Chicago, IL, United States). Descriptive statistics were reported as means ± standard deviation (M±SD). Normality of all variables was confirmed using the Shapiro-Wilk test. Baseline between-group differences were assessed using one-way analysis of variance (ANOVA). Differences among the SS, DS, and NS groups across the tests (SBT, YBT, CoD T-test, CMJT, SJT, and FJT) were analyzed using two-factor repeated measures ANOVA, which examined both the main effects of time and the interaction effects between time and group. Bonferroni adjustment was applied for multiple comparisons. A significance level of *P* < 0.05 was used.

## 3 Results

The M ± SDs and changes in performance assessment are displayed in Tables 3, 4. The Shapiro-Wilk tests indicated that all groups’ data followed a normal distribution. The subsequent analysis used one-way repeated measures ANOVA, and the results showed significant main effects of time for SBT (right: F = 127.97, P < 0.05; left: F = 139.32, P < 0.05), YBT (right/AT: F = 12.68, P < 0.05; right/PM: F = 70.25, P < 0.05; right/PL: F = 18.96, P < 0.05; left/AT: F = 28.98, P < 0.05; left/PM: F = 60.97, P < 0.05; left/PL: F = 87.30, P < 0.05), and CoD T-test (F = 43.38, P < 0.05). Additionally, significant interaction effects between time and group were observed for SBT (right: F = 29.39, P < 0.05; left: F = 27.75, P < 0.05), YBT (right/AT: F = 18.41, P < 0.05; right/PM: F = 61.51, P < 0.05; right/PL: F = 31.13, P < 0.05; left/AT: F = 29.79, P < 0.05; left/PM: F = 48.15, P < 0.05; left/PL: F = 33.21, P < 0.05), CoD T-test (F = 44.49, P < 0.05), and CMJ (F = 8.70, P < 0.05) (Table 3).

**TABLE 3:** Measures and comparisons of variables before (pre-test) and after (post-test) training program across different groups (SS, DS, NS).

Variables			SS (n = 20)		DS (n = 20)		NS (n = 20)		F-values (*P*-values)	
			Pre	Post	Pre	Post	Pre	Post	Time	Time*Group
SBT (s)	R		18.63 ± 1.27	22.17 ± 1.02	18.84 ± 1.02	22.51 ± 1.28	19.19 ± 0.81	19.29 ± 1.23	127.97 **(0.00)**	29.39 **(0.00)**
	L		18.87 ± 0.81	22.63 ± 1.28	19.07 ± 0.83	22.94 ± 1.43	18.95 ± 1.22	19.24 ± 1.05	139.32 **(0.00)**	27.75 **(0.00)**
YBT (cm)	R	*AT*	89.85 ± 3.42	87.95 ± 2.56	89.15 ± 3.17	96.25 ± 3.82	87.45 ± 3.00	88.80 ± 3.37	12.68 **(0.00)**	18.41 **(0.00)**
		*PM*	98.80 ± 2.46	97.85 ± 3.07	98.85 ± 3.28	111.25 ± 3.14	98.90 ± 2.20	100.55 ± 2.26	70.25 **(0.00)**	61.51 **(0.00)**
		*PL*	58.65 ± 1.60	58.10 ± 1.71	57.55 ± 1.47	64.20 ± 1.51	58.70 ± 3.03	58.20 ± 3.90	18.96 **(0.00)**	31.13 **(0.00)**
	L	*AT*	88.85 ± 3.23	89.40 ± 2.23	89.05 ± 3.38	98.35 ± 3.96	89.80 ± 2.46	89.20 ± 2.82	28.98 **(0.00)**	29.79 **(0.00)**
		*PM*	99.35 ± 2.37	98.60 ± 2.58	99.45 ± 3.94	112.45 ± 4.19	98.90 ± 4.72	100.85 ± 3.66	60.97 **(0.00)**	48.15 **(0.00)**
		*PL*	58.25 ± 1.33	58.80 ± 2.09	58.95 ± 1.32	65.00 ± 1.30	58.50 ± 1.76	60.10 ± 1.77	87.30 **(0.00)**	33.21 **(0.00)**
CoD T-test (s)			6.61 ± 0.05	6.62 ± 0.04	6.64 ± 0.05	6.48 ± 0.04	6.63 ± 0.02	6.62 ± 0.04	43.38 **(0.00)**	44.49 **(0.00)**
CMJ (cm)			39.18 ± 4.422	37.15 ± 4.83	39.44 ± 4.23	44.49 ± 2.92	38.83 ± 3.66	37.74 ± 3.12	0.74 (0.39)	8.70 **(0.00)**
SJ (cm)			36.37 ± 2.40	36.76 ± 2.19	37.40 ± 2.36	38.95 ± 1.87	37.55 ± 2.38	36.52 ± 2.71	0.47 (0.50)	2.73 (0.07)
FJT (cm)			7.87 ± 0.52	7.87 ± 0.53	7.98 ± 0.50	8.20 ± 0.47	7.97 ± 0.33	8.04 ± 0.52	1.09 (0.30)	0.50 (0.61)

The meaning of bold values was p < 0.05.

**TABLE 4:** Intra group comparisons of variables before (pre-test) and after (post-test) training program.

Variables			SS (n = 20)		DS (n = 20)		NS (n = 20)	
			Mean change	*P*-values	Mean change	*P*-values	Mean change	*P*-values
SBT (s)	R		3.54 ± 0.37	**0.00**	3.66 ± 0.37	**0.00**	0.10 ± 0.37	0.79
	L		3.75 ± 0.39	**0.00**	3.87 ± 0.39	**0.00**	0.28 ± 0.39	0.47
YBT (cm)	R	*AT*	−1.90 ± 1.06	0.08	7.10 ± 1.06	**0.00**	1.35 ± 1.06	0.21
		*PM*	−0.95 ± 0.90	0.30	12.40 ± 0.90	**0.00**	1.65 ± 0.90	0.07
		*PL*	−0.55 ± 0.74	0.46	6.65 ± 0.74	**0.00**	−0.50 ± 0.74	0.50
	L	*AT*	0.55 ± 0.99	0.58	9.30 ± 0.99	**0.00**	−0.60 ± 0.99	0.55
		*PM*	−0.75 ± 1.05	0.48	13.00 ± 1.05	**0.00**	1.95 ± 1.05	0.07
		*PL*	0.55 ± 0.51	0.28	6.05 ± 0.51	**0.00**	1.60 ± 0.51	**0.00**
CoD T-test (s)			0.01 ± 0.01	0.57	−0.16 ± 0.01	**0.00**	−0.01 ± 0.01	0.62
CMJ (cm)			−2.02 ± 1.30	0.13	5.05 ± 1.30	**0.00**	−1.09 ± 1.30	0.41
SJ (cm)			0.39 ± 0.77	0.62	1.55 ± 0.77	**0.05**	1.02 ± 0.77	0.19
FJT (cm)			−0.00 ± 0.16	0.99	0.22 ± 0.16	0.18	0.07 ± 0.16	0.65

The meaning of bold values was p < 0.05.

Intra-group comparisons revealed that, compared to pre-training, the SS group showed a significant increase in SBT duration after training, with statistical significance (P < 0.05). However, variables such as YBT, CoD T-test, CMJ, SJ, and FJT did not show statistically significant differences. In the DS group, compared to pre-training, significant improvements were observed in SBT duration, YBT distances in all directions, CMJ and SJ heights, as well as CoD T-test duration, all with statistical significance (P ≤ 0.05). The SS group did not show significant improvement in FJT. Additionally, the NS group showed a significant improvement in YBT in the left leg’s PL direction (P < 0.05), with no statistically significant differences observed in other variables (Table 4).

Baseline comparisons showed no significant differencea in any variables among the three groups before the training program (Table 5). Subsequent inter-group comparisons post-training revealed that compared to the DS group, the SS group exhibited significant decreases in YBT distances, CMJ and SJ heights, and a significant increase in CoD T-test duration, all with statistical significance (P < 0.05). Compared to the NS group, the SS group showed a significant increase in SBT duration and a significant decrease in YBT distance in the right leg’s PM direction, with statistical significance (P < 0.05). Compared to the NS group, the DS group demonstrated statistically significant improvements in all variables (P < 0.05) except FJT, namely significant increases in SBT duration, YBT distances, CMJ and SJ heights, and a significant decrease in CoD T-test duration (Table 6).

**TABLE 5:** Pairwise comparisons between groups of variables before (pre-test) training program.

Variables			SS vs. DS		SS vs. NS		DS vs. NS	
			Mean change	*P*-values	Mean change	*P*-values	Mean change	*P*-values
SBT (s)	R		−0.21 ± 0.33	1.00	−0.56 ± 0.33	0.30	−0.34 ± 0.33	0.92
	L		−0.20 ± 0.31	1.00	−0.08 ± 0.31	1.00	0.12 ± 0.31	1.00
YBT (cm)	R	*AT*	0.07 ± 1.01	1.00	2.40 ± 1.01	0.06	1.70 ± 1.01	0.30
		*PM*	−0.05 ± 0.85	1.00	−0.10 ± 0.85	1.00	−0.05 ± 0.85	1.00
		*PL*	1.10 ± 0.68	0.33	−0.05 ± 0.68	1.00	−1.15 ± 0.68	0.30
	L	*AT*	−0.20 ± 0.97	1.00	−0.95 ± 0.97	0.99	−0.75 ± 0.97	1.00
		*PM*	−0.10 ± 1.20	1.00	0.45 ± 1.20	1.00	0.55 ± 1.20	1.00
		*PL*	−0.07 ± 0.47	0.43	−0.25 ± 0.47	1.00	0.45 ± 0.47	1.00
CoD T-test (s)			−0.03 ± 0.01	0.15	−0.02 ± 0.01	0.58	0.01 ± 0.01	1.00
CMJ (cm)			−0.26 ± 1.30	1.00	0.34 ± 1.30	1.00	0.61 ± 1.30	1.00
SJ (cm)			−1.04 ± 0.75	0.52	−1.18 ± 0.75	0.37	−0.15 ± 0.75	1.00
FJT (cm)			−0.11 ± 0.15	1.00	−0.10 ± 0.15	1.00	0.01 ± 0.15	1.00

**TABLE 6:** Pairwise comparisons between groups of variables after (post-test) training program.

Variables			SS vs. DS		SS vs. NS		DS vs. NS	
			Mean change	*P*-values	Mean change	*P*-values	Mean change	*P*-values
SBT (s)	R		−0.34 ± 0.37	1.00	2.88 ± 0.37	**0.00**	3.22 ± 0.37	**0.00**
	L		−0.32 ± 0.40	1.00	0.39 ± 0.40	**0.00**	3.70 ± 0.40	**0.00**
YBT (cm)	R	*AT*	−8.30 ± 1.04	**0.00**	−0.85 ± 1.04	1.00	7.40 ± 1.04	**0.00**
		*PM*	−13.40 ± 0.90	**0.00**	−2.70 ± 0.90	**0.01**	10.70 ± 0.90	**0.00**
		*PL*	−6.10 ± 0.83	**0.00**	−0.10 ± 0.83	1.00	6.00 ± 0.83	**0.00**
	L	*AT*	−8.95 ± 0.98	**0.00**	0.20 ± 0.98	1.00	9.15 ± 0.98	**0.00**
		*PM*	−13.85 ± 1.12	**0.00**	−2.25 ± 1.12	0.15	11.60 ± 1.12	**0.00**
		*PL*	−6.20 ± 0.55	**0.00**	−1.30 ± 0.55	0.07	4.90 ± 0.55	**0.00**
CoD T-test (s)			0.14 ± 0.01	**0.00**	−0.00 ± 0.01	1.00	−0.14 ± 0.01	**0.00**
CMJ (cm)			−7.34 ± 1.18	**0.00**	−0.59 ± 1.18	1.00	6.75 ± 1.18	**0.00**
SJ (cm)			−2.20 ± 0.72	**0.01**	0.23 ± 0.72	1.00	2.43 ± 0.72	**0.00**
FJT (cm)			−0.33 ± 0.16	0.13	−0.18 ± 0.16	0.82	0.16 ± 0.16	1.00

The meaning of bold values was p < 0.05.

## 4 Discussion

This study demonstrated that (i) both SS and DS significantly enhance SBT, with no significant difference in their effects, (ii) only DS significantly improved YBT in all directions, and (iii) only DS significantly improved neuromuscular performance indicators such as CoD T-test, CMJ, and SJ. It can be seen that among healthy and active men, both SS and DS positively impact static balance ability. DS also shows beneficial effects on dynamic balance ability and neuromuscular performance, including agility and jumping ability, whereas SS does not.

This study reported the positive impact of SS on static balance. However, contrasting findings from Behm et al. and Chatzopoulos D et al. indicated a negative impact of SS on balance performance (Behm et al., 2004; Chatzopoulos et al., 2014). Another study by Coratella et al. (2021b) suggested that SS does not affect balance when muscle activation is increased. One possible explanation for these difference could be the varying duration of stretching in their respective training protocols. In our study, each muscle group was stretched for 1-2 sets of 30 s, whereas Behm et al. employed 3 sets of 45-s stretches (Behm et al., 2004). Prolonged SS may decreases muscle tendon unit stiffness (Konrad and Tilp, 2014) and compromise muscle balance performance. Another possible explanation is the differences in gender and age among the research subjects. Our study focused on adult male college students, whereas Chatzopoulos D et al. examined adolescent females (Chatzopoulos et al., 2014). Variations in muscle stiffness and viscosity between these groups may influence their responses to stretching techniques. Moreover, this study observed a significant positive impact of DS on dynamic and static balance, suggesting superior balance performance enhancement compared to SS. This observation can be attributed to two key factors. Firstly, DS enhances muscle temperature (Fletcher and Jones, 2004). The rhythmic contraction and stretching movements during DS effectively raise muscle temperature and overall body warmth. This rise in temperature contributes to reduced muscle viscosity, thereby enhancing muscle elasticity and promoting better balance performance. Secondly, DS exerts stimulatory effects on the nervous system (Jaggers J. R. et al., 2008). Studies have demonstrated that DS increases electromyographic amplitude, indicating greater muscle activation and neuromuscular efficiency (Herda et al., 2008). This heightened activation is crucial for improving neuromuscular coordination and responsiveness, which are essential components of enhanced balance performance. Additionally, DS may enhance neural drive to the muscles, increasing the speed and accuracy of neuromuscular responses during postural adjustments (Jaggers R. R. et al., 2008). The result is improved muscle activation and faster reflexes, enabling individuals to respond more effectively to balance challenges and thereby enhancing both static and dynamic balance.

In this study, SS did not demonstrate any positive effects on neuromuscular adaptation, which aligns with the findings of Blazevich A J et al. and Yapicioglu B et al. (Fortier et al., 2013; Blazevich et al., 2018). In contrast, Damasceno MV et al. and Kilit B et al. reported different results, indicating that SS may impair agility and jumping performance (Damasceno et al., 2014; Kilit et al., 2018). The differing outcomes could be attributed to variations in stretching protocols, particularly the duration of static stretches for each muscle group. For instance, similar to our study, Blazevich AJ et al. and Yapicioglu B et al. employed 30-s static stretches and found no significant effects on CMJ, VJ, and sprint speed (Fortier et al., 2013; Blazevich et al., 2018). However, Damasceno MV observed a notable 9.2% decrease in CMJ following a 90-s stretch (Damasceno et al., 2014), whereas Kilit B et al. reported reduced sprint times after a 60-s stretch (Kilit et al., 2018). A comparative investigation by Pinto MD et al. revealed a 3.4% decrease in CMJ after 60 s of static stretching, whereas no statistically significant effect was observed with a 30-s stretch (Pinto et al., 2014). These findings collectively indicate that SS of each muscle group for more than 30 s may indeed have adverse effects on neuromuscular adaptability. Furthermore, in this study, DS significantly enhanced jumping performance, underscoring its positive impact on neuromuscular adaptation. Numerous studies have similarly reported these findings (Perrier et al., 2011; Kruse et al., 2013; Ryan et al., 2014; Kruse et al., 2015). For instance, KruseNT et al. observed a significant increase in CMJ height among female athletes following DS (Kruse et al., 2013; Kruse et al., 2015); and Perrier et al. (2011) demonstrated that dynamic stretching significantly improved VJ height in active males. These positive effects may be related to the movement pattern of DS, which stimulates muscle spindles, enhances muscle reflex activity, and contributes to increased strength and power (Perrier et al., 2011; Alemdaroglu et al., 2017). Additionally, DS likely activates the nervous system, leading to improved performance as evidenced by a notable increase in electromyographic amplitude following DS interventions (Fletcher, 2010). This suggests enhanced motor unit recruitment within the neuromuscular system after DS. By enhancing signal transmission between the brain, spinal cord, and muscles, DS improves neuromuscular efficiency, reflex speed, and adaptability to specific movements. Recent studies also show that DS engages both the peripheral and central nervous systems, enhancing neuromuscular coordination and motor response speed (Avela et al., 2006).

One limitation of this study is its use of an intergroup design instead of an intragroup crossover design. While an intragroup crossover design can mitigate the influence of individual differences or variability between trial periods on study outcomes, it also carries the risk of a learning effect in the tests. This effect could arise from subjects being asked to return on three separate occasions to perform each stretching regime. In contrast, an intergroup design avoids this issue and requires less time investment.

## 5 Conclusion

The present results indicate that SS demonstrates positive acute effects solely on static balance adaptation, while DS exhibits positive acute effects on both neuromuscular and balance adaptations.

## Data Availability

The raw data supporting the conclusions of this article will be made available by the authors, without undue reservation.
